# A novel nerve transection and repair method in mice: histomorphometric analysis of nerves, blood vessels, and muscles with functional recovery

**DOI:** 10.1038/s41598-020-78481-1

**Published:** 2020-12-10

**Authors:** Jung Il Lee, Anagha A. Gurjar, M. A. Hassan Talukder, Andrew Rodenhouse, Kristen Manto, Mary O’Brien, Prem Kumar Govindappa, John C. Elfar

**Affiliations:** 1 Department of Orthopaedics and Rehabilitation, Center for Orthopaedic Research and Translational Science, The Pennsylvania State University College of Medicine, Milton S. Hershey Medical Center, 500 University Drive, Mail Code H089, Hershey, PA 17033 USA; 2 Department of Orthopedic Surgery, Hanyang University College of Medicine, Hayang University Guri Hospital, Guri, South Korea

**Keywords:** Physiology, Medical research

## Abstract

Peripheral nerve transection is associated with permanent functional deficit even after advanced microsurgical repair. While it is difficult to investigate the reasons of poor functional outcomes of microsurgical repairs in humans, we developed a novel pre-clinical nerve transection method that allows reliable evaluation of nerve regeneration, neural angiogenesis, muscle atrophy, and functional recovery. Adult male C57BL/6 mice were randomly assigned to four different types of sciatic nerve transection: *Simple Transection *(*ST*),* Simple Transection & Glue *(*TG*), *Stepwise Transection and Sutures *(*SU*), and *Stepwise Transection and Glue *(*STG*). Mice were followed for 28 days for sciatic function index (SFI), and sciatic nerves and hind limb muscles were harvested for histomorphological and cellular analyses. Immunohistochemistry revealed more directional nerve fiber growth in SU and STG groups compared with ST and TG groups. Compared to ST and TG groups, optimal neural vessel density and branching index in SU and STG groups were associated with significantly decreased muscle atrophy, increased myofiber diameter, and improved SFI. In conclusion, our novel STG method represents an easily reproducible and reliable model with close resemblance to the pathophysiological characteristics of SU model, and this can be easily reproduced by any lab.

## Introduction

Peripheral nerve injury represents a major clinical and public health problem that often leads to significant functional impairment and permanent disability^1^. It is estimated that roughly 3% of all trauma patients have peripheral nerve injuries^2^ and more than 50,000 peripheral nerve repair procedures are performed annually in the United States alone^3^. Peripheral nerve injury occurs along a spectrum from injuries in which some axonal continuity is maintained and injuries involving complete nerve transection^4–6^. In those injuries where continuity is maintained (such as traumatic crush or compression injuries), axonal regeneration is often successful and near optimal functional recovery may be seen within few weeks of injury. However, the situation is different with transection injury where two ends of the severed nerve are separated by small intervening gap. Currently the treatment of choice for nerve transection injury is advanced microsurgical end-to-end repair with tensionless epineurial sutures or autologous nerve grafting if end-to-end anastomosis is not possible^7,8^. Despite these highly advanced microsurgical and reconstructive techniques, the functional recovery after peripheral nerve repair is often unsatisfactory, but these approaches often fail to address the complex cellular and molecular events associated with peripheral nerve injury and repair^9,10^.

Axonal misdirection, inadequate axonal regeneration, and delayed muscle reinnervation contribute significantly to the poor functional outcome and therapeutic failure after nerve repair^11–16^. Angiogenesis at the site of transection is also reported to play a significant role in nerve regeneration^17,18^. While it is difficult to investigate the mechanisms of poor functional outcomes in humans, the impact of microsurgical techniques also remains largely unknown. Importantly, microscopic manipulations can cause additional trauma/damage by excessive mobilization or manipulation of the nerve or by frequent needle use^19^. The disadvantages with micro-suturing may confound outcomes when investigating the effect of new therapeutic strategy. Therefore, there is an unmet need for a novel experimental nerve transection model with small intervening gap that would allow reliable evaluation of the pathophysiology of nerve injury, cellular and molecular mechanisms of nerve regeneration, neural vascularization, muscle atrophy, and functional recovery.

The hypothesis we first set out to test was that perfectly aligned transected nerve ends with absolute and rigorous prevention of stump retraction, would recover function. We also wanted to test the related hypothesis that, in such a model, where the nerve was ensured to be aligned and approximated, that the muscle atrophy would, correlate to the rigor of the repair. Finally we chose a standard repair of severed nerves with suture for comparison with such an idealized repair, along with a simple laceration left either unrepaired or repaired with fibrin glue placement. Fibrin glue is widely used in laboratory research and clinical nerve repair scenarios^20,21^. Several studies have shown that fibrin glue can be an excellent alternative to epineural sutures in nerve repair^19,22,23^. Fibrin glue is easy to use, takes less surgical time, and causes less local inflammation and scarring. Therefore, we used fibrin glue in a novel step-wise nerve transection model in addition to the gold-standard epineurial micro-suturing model.

In this study, we present a novel standardized peripheral nerve transection method in mice using fibrin glue for modeling peripheral nerve transection injury with reproducible gap distance between the severed nerve ends. The main objective of this study was to evaluate the morphological and quantitative histomorphometric characteristics of peripheral nerves, neural microvessels, and skeletal muscles, as well as the functional recovery in four different types of nerve transection models.

## Results

### Propensity of misdirectional nerve regeneration in different transection models

**Figure 1:**
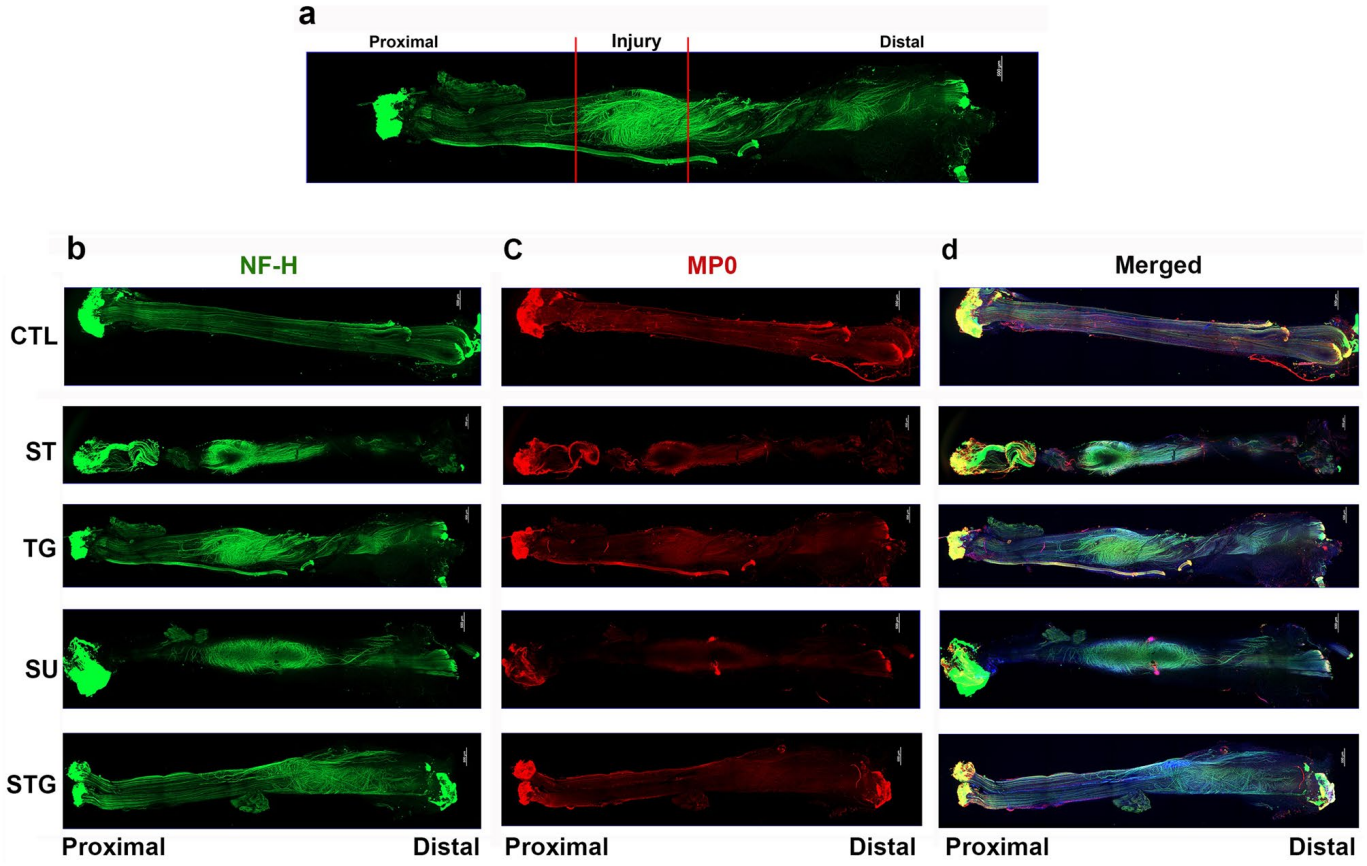
Evaluation of whole mount immunostaining of the transected nerve at post-injury day 28 for nerve fiber distribution. (**a**) For the purpose of evaluation and quantification, imaged whole nerve was divided into 3 zones: proximal, injury and distal. Representative compact images of immunofluorescence staining of the whole nerve for NF-H (**b**) in green, MP0 (**c**) in red, and the merged view (**d**). Scale bar, 500 µm (at upper right corner); magnification, × 5. For other details, see Fig. 8.

At post-injury day 28, all nerves were found in good continuity with a bulge near the transection site and there was no dehiscence with any repair method. Misdirection of the regenerating axons is considered one of the major critical factors in poor functional outcome after nerve injury and repair^11,12,16^. To evaluate the distribution, direction, and alignment of regenerating nerve filaments, immunofluorescence staining of the whole nerve was performed for NF-H and MP0. Figure 1a outlines the zoning of the transected nerve as described in the Method. Uninjured nerve displays normal nerve architecture with uniform NF-H (Fig. 1b), MP0 (Fig. 1c) and merged (Fig. 1d) stainings over the entire length, and unidirectional nerve fibers are compactly packed and parallelly aligned. Neurofilaments which run parallel to proximal–distal axis are vertical filaments. In all transection models, axons from proximal stump tried to grow towards the distal end through the repair site and led to a bulging at the nerve bridge. Compared to the STG, NF-H intensity in the injury zone of ST, TG, and SU groups was more pronounced probably because of increased number of misdirected nerve fibers.

During surgery, ST and TG groups had a wider gap between the proximal and distal nerve stumps, whereas SU and STG groups had a minimum gap between the severed nerve ends because of closer approximation. NF-H staining in the distal zone of ST group revealed a low intensity compared to progressively increased NF-H intensity in the distal zone of other groups (TG < SU < STG), indicating that closer approximation of severed nerve ends enables greater number of axons to cross the gap towards the distal end. MP0 staining in the injury and distal zones of all groups displayed intensities comparable to that of NF-H staining. The STG had closest approximation of aligned nerve ends with ideally opposed nerve fascicles. Thus, as compared to other groups, we observed greater numbers of compactly packed, parallelly aligned myelinated axons in both proximal and distal zones of STG group. Supplementary Fig. 1a shows the representative compact images of negative control for NF-H and MP0.

**Figure 2:**
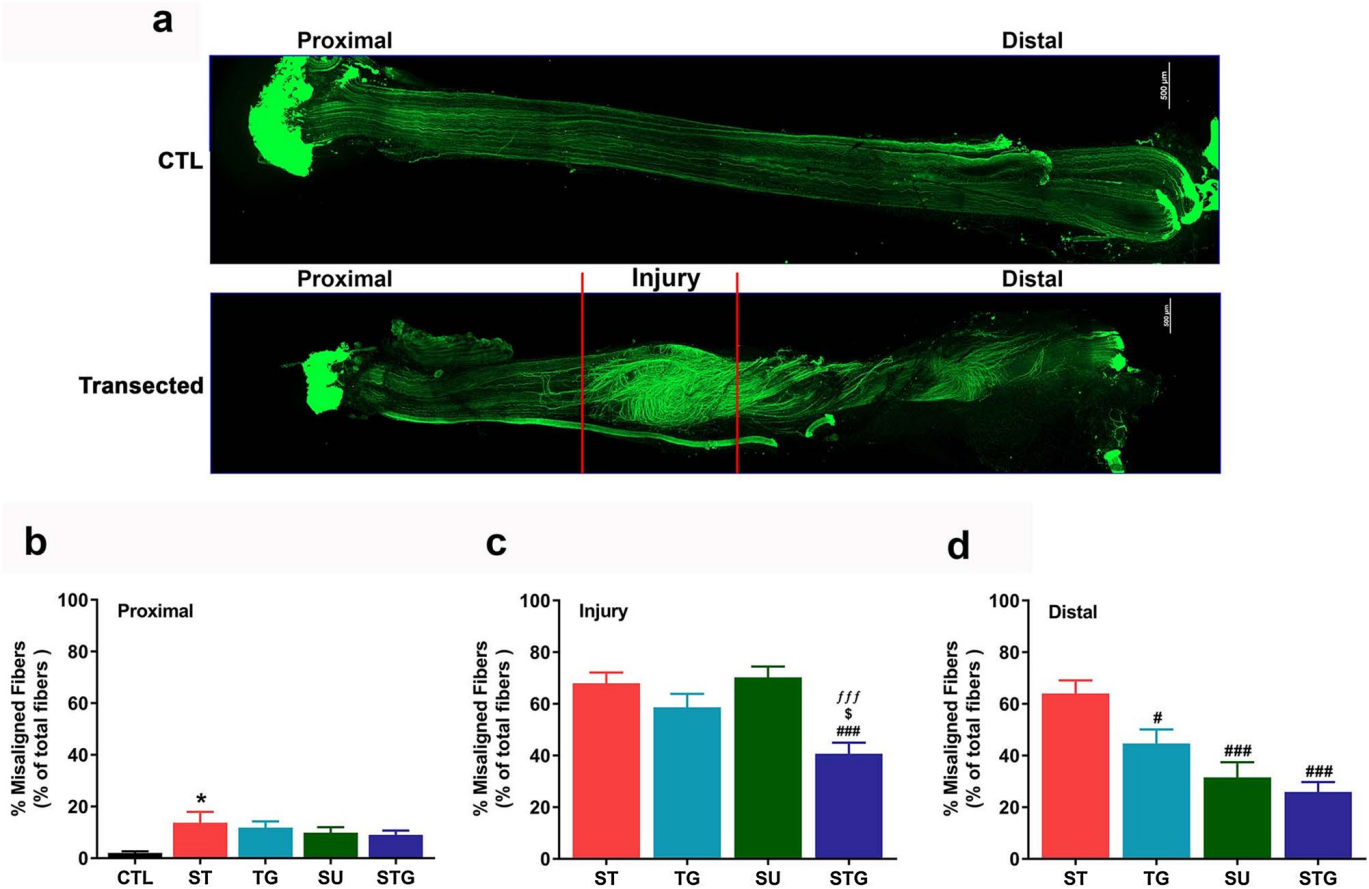
(**a**) Representative images from an uninjured and a transected nerve to show the orientation of neurofilaments at different zones. Scale bar, 500 µm (at upper right corner). Bar graph showing the quantification of misaligned fibers at proximal (**b**), injury (**c**) and distal (**d**) zones of transected nerves. Misaligned fibers are shown as the percentage of total number of fibers in each zone. n = 3/group, *P < 0.05, ***P < 0.001 vs. uninjured (CTL), ^#^*P* < 0.05, ^###^*P* < 0.001 vs. ST, ^$^*P* < 0.05 vs. TG, and ^ƒƒƒ^*P* < 0.001 vs. SU. For other details, see Fig. 8.

To evaluate the extent of misdirection, we quantified nerve fiber alignment in different zones. Figure 2a shows the patterns of nerve fiber distribution as described above in high resolution at different zones of an uninjured (top) and transected (bottom) nerves. Figure 2b, c, d shows quantitative analysis. Compared to the uninjured nerve, the number of misaligned nerve fibers in all groups were markedly higher in all three zones. While ST had significantly highest ratio of misaligned nerve fibers in the proximal zone (Fig. 2b), ST, TG, and SU groups had higher ratio of misaligned nerve fibers in the injury zone (Fig. 2c). In the distal zone (Fig. 2d), the ratio of misaligned fibers was highest in the ST group and lowest in the STG group. Importantly, misaligned fibers in the distal zone progressively decreased from ST to STG group (ST > TG > SU > STG). These findings suggest that irrespective of the orientation of nerve fascicles, a close approximation of severed nerve ends reduces nerve fiber misalignment during nerve regeneration.

### Angiogenesis in different nerve transection models

**Figure 3:**
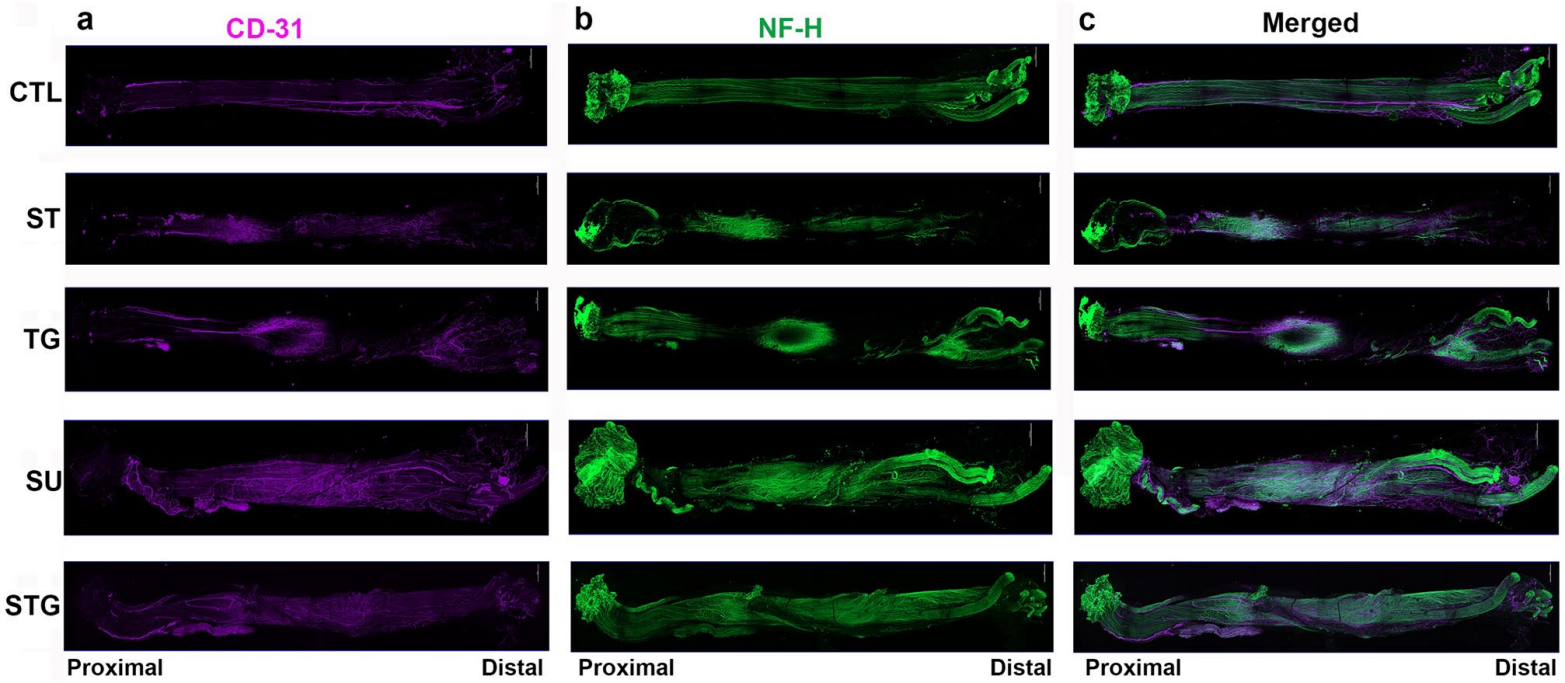
Evaluation of whole mount immunostaining of the transected nerve at post-injury day 28 for angiogenesis. Representative compact images of immunofluorescence staining of the whole nerve for CD31 (**a**) in purple, NF-H (**b**) in green, and the merged view (**c)**. Scale bar, 500 µm (at upper right corner); magnification, × 5. For other details, see Fig. 8.

**Figure 4:**
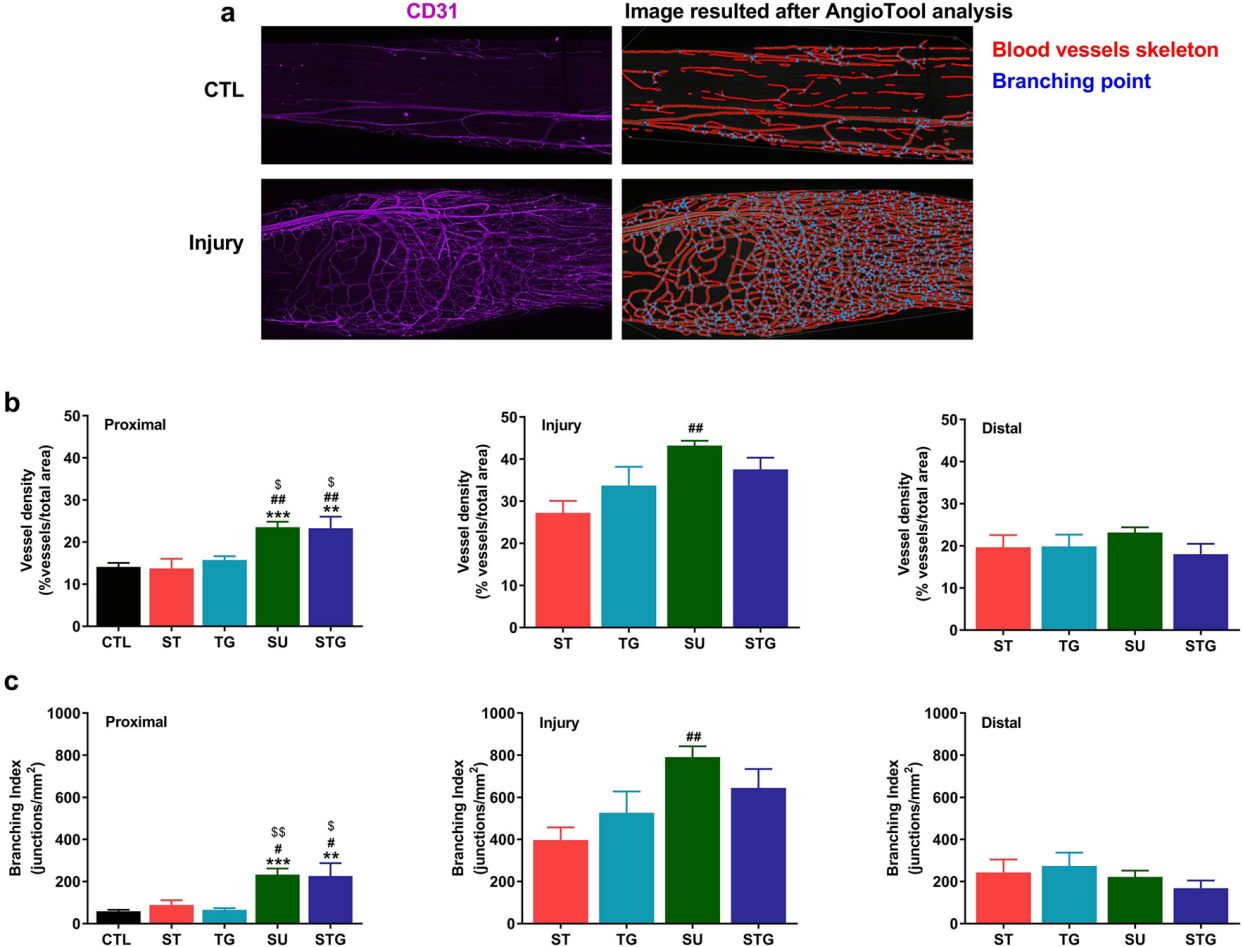
(**a**) Representative images from an uninjured and a transected nerve to show the blood vessels (left) and AngioTool reconstruction result (right) images. AngioTool images clearly depict the blood vessel architecture as red lines and their branching points as blue dots. Bar graph showing the quantification of blood vessels at proximal, injury, and distal zones of transected nerves. (**b**) Blood vessel density is shown as the percentage of number of blood vessel in total area. (**c**) Branching index of blood vessels is shown as the number of blood vessel junctions/mm^2^. n = 3/group, **P < 0.01, ***P < 0.001 vs. uninjured (CTL), ^#^*P* < 0.05, ^##^*P* < 0.01 vs. ST, and ^$$^*P* < 0.05 vs. TG. For other details, see Fig. 8.

Angiogenesis plays an important role in axonal sprouting, regeneration and reinnervation following nerve transection injuries^17,18,24^. Using the endothelial cell marker CD31, we examined new blood vessel formation in different transection modes at post-injury day 28. Figure 3 displays the immunofluorescence staining of the whole nerves with CD31, NF-H, and their merged images. In the uninjured nerves, immunofluorescence staining was uniform over the entire length. In contrast, in transected nerves, CD31 staining intensity in the injury zone was more pronounced in all groups compared to their respective proximal and distal zones. Figure 4a shows the representative blood vessels (left) and AngioTool result (right) images of uninjured and transected nerves. AngioTool images clearly depict the blood vessel architecture (red lines) and their bracnching points (blue dots). Quantification of blood vessel density (Fig. 4b) and branching index (Fig. 4c) also revealed that injury zone had a higher blood vessel density and branching index than proximal and distal zones. In the injury zone, blood vessel density and branching index progressively increased from ST to STG group (ST < TG < SU ≥ STG), and the SU group was significantly different from the ST group. Interestingly, blood vessel density and branching index in the proximal zone were significantly higher in SU and STG groups compared to uninjured, ST or TG group, but there was no significant difference between these groups in the distal zone. Of note, blood vessel density and branching index levels in SU and STG groups were relatively comparable between proximal and distal zones, indicating a directional angiogenesis in these groups. Supplementary Fig. 1b shows the representative compact images of negative control for NF-H and CD31.

### Muscle atrophy in different nerve transection models

**Figure 5:**
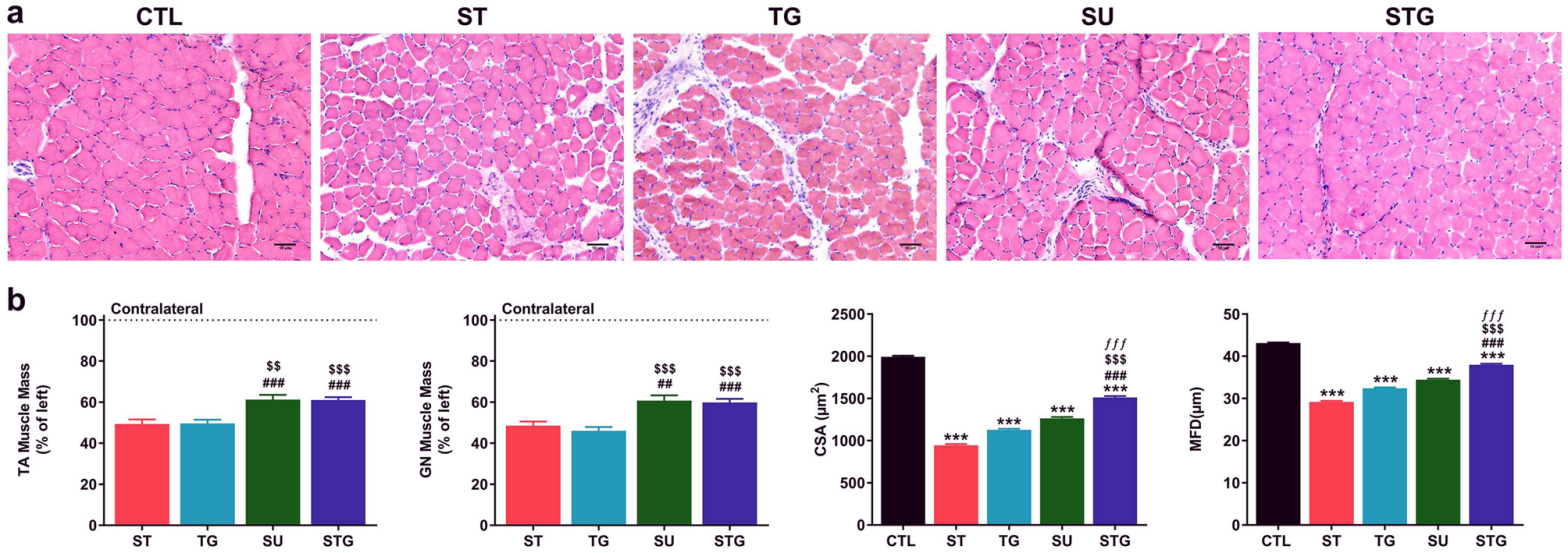
Effect of different transection models on TA muscle histology, muscle mass, and quantitative measurements of the muscle fibers. (**a**) Representative images of TA muscle cross sections stained with H&E. Scale bar, 50 µm (at lower right corner); magnification 20 ×. (**b**) TA muscle mass as % of left side, n = 10–12/group; CSA of TA muscle as µm^2^, n = 3/group; and MFD of TA muscle as µm, n = 3/group. ***P < 0.001 vs. CTL, ^###^*P* < 0.001 vs. ST, ^$$^*P* < 0.01, ^$$$^*P* < 0.001 vs. TG, and ^ƒƒƒ^*P* < 0.001 vs. SU. For other details, see Fig. 8.

While innervation of skeletal muscle is essential for the maintenance of muscle size, structure, contractile function, and mobility, denervation results in rapid muscle-fiber atrophy^14,15,25^. To evaluate muscle atrophy in different transection methods, the histological and histomorphometric analysis of muscles were performed. *First,* we checked the changes in muscle mass at post-injury day 28 and Fig. 5a displays representative H&E images of frozen transverse sections of TA muscle from different groups (Fig. 5a). Uninjured TA muscle shows normal muscle architecture with a tightly packed homogeneous polygonal shaped muscle fiber distribution, peripherally placed nuclei, minimal intramyofiber spacing, and little cellular infiltration. Compared to uninjured muscle, transverse sections of ST, TG, and SU groups show muscle fibers with increased intermyofiber spacing, cellular infiltration, and fibrotic changes. STG, on the contrary, displays tightly packed myofibers of uniform size and shape with minimal intramyofiber spacing, cellular infiltrations, and fibrotic changes.

Quantitative analysis in Fig. 5b revealed that the gross muscle weight in injured right limb was markedly reduced compared to contralateral healthy left limb, and muscle atrophy was more pronounced in ST and TG groups. Right TA muscle weight (as % of contralateral left side) in ST, TG, SU, and STG groups were 49.4 ± 2.15%, 49.6 ± 1.73%, 61.2 ± 2.31%, and 61.0 ± 1.39%, respectively. It is apparent that the decrease in muscle weight was proportional to the gap distance between the severed ends of transected nerve because ST and TG groups had greater muscle loss compared to SU and STG groups. Next, to further address the muscle loss in relation to the gap distance between severed nerve ends, we performed histomorphometric analysis of muscle cross sections to quantify CSA and MFD in all groups. Quantitative analysis revealed significant decrease in both CSA and MFD in all the groups as compared to uninjured group (1993.45 ± 12.90 µm^2^, 43.12 ± 0.15 µm). CSA and MFD values progressively increased from ST (944.23 ± 14.42 µm^2^, 29.18 ± 0.23 µm), TG (1127.82 ± 12.46 µm^2^, 32.42 ± 0.20 µm), SU (1263.71 ± 16.04 µm^2^, 34.43 ± 0.24 µm) to STG (1511.54 ± 17.33 µm^2^, 38.00 ± 0.24 µm) group. Of note, STG group showed significantly large CSA and MFD as compared to SU and other groups indicating minimum muscle atrophy with novel nerve transection method.

**Figure 6:**
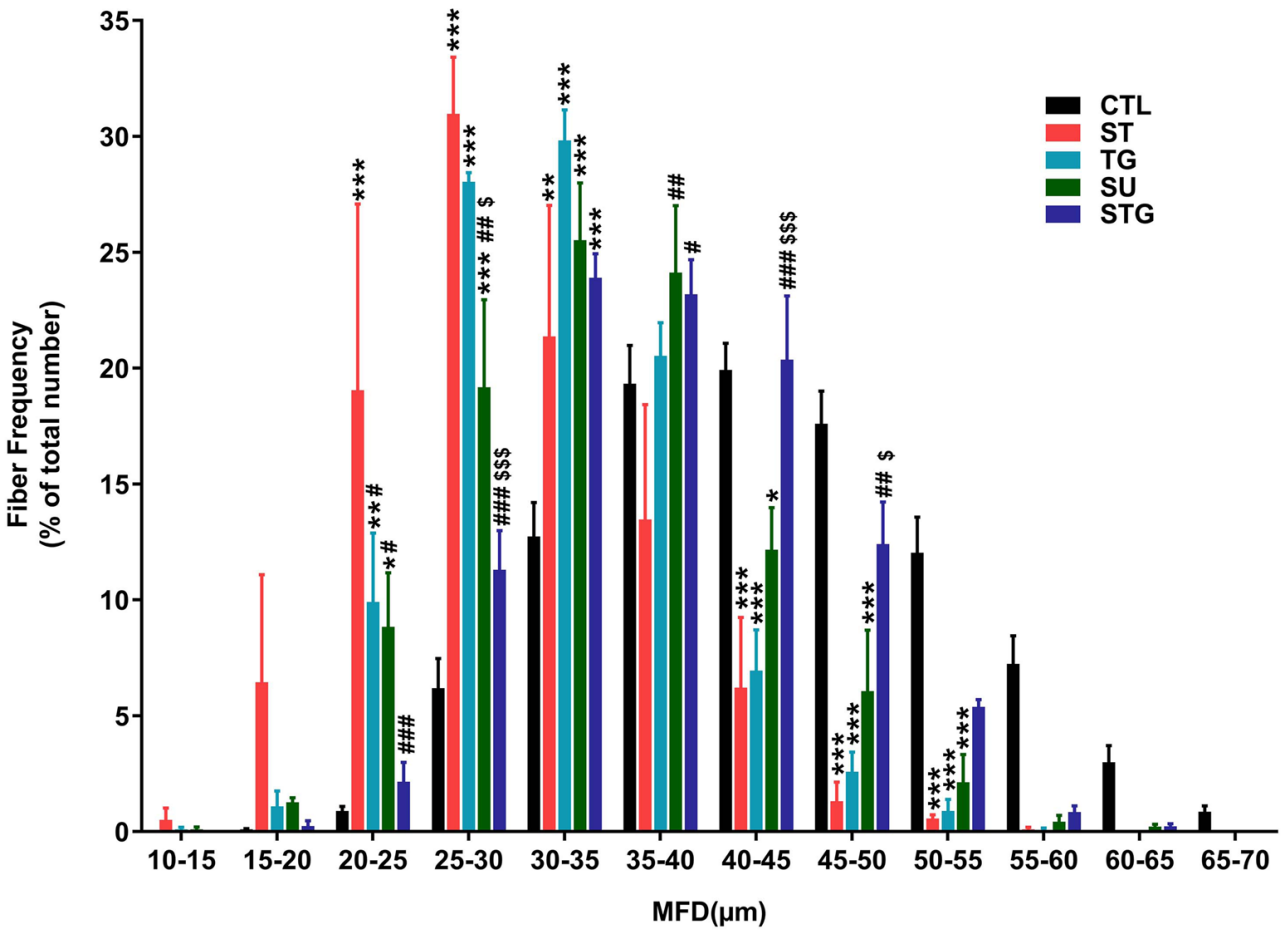
Effect of different transection models on muscle fiber distribution. Quantitative muscle fiber size distribution from the MFD value (µm) in each group as a percentage of total fiber number. n = 3/group; *P < 0.05, **P < 0.01, ***P < 0.001 vs. uninjured (CTL), ^#^*P* < 0.05, ^##^*P* < 0.01, ^###^*P* < 0.001 vs. ST, and ^$$$^*P* < 0.001 vs. TG. For other details, see Fig. 8.

Finally, we analyzed muscle fiber distribution (Fig. 6) based on the MFD data which revealed that 69% fibers in the uninjured group were distributed evenly in a range 35–55 µm, other 11% had higher MFD (55–70 µm) and only 20% fibers were smaller (< 35 µm). From the 20% smaller fibers, maximum (13%) were within MFD of 30–35 µm. The ratio of muscle fiber within 35–55 µm ranges in ST, TG, and SU group was 21%, 31%, and 44%, respectively. The ratio of smaller fibers (< 35 µm) in ST, TG, and SU group was 78%, 68%, and 54%, respectively. Histograms for ST, TG, and SU groups thus revealed a left shift in fiber distribution with predominance of smaller fibers. Similar to uninjured nerve, STG group had 61% fibers in a range 35-55 µm and 37% fibers were smaller fibers (< 35 µm). Furthermore, 24% of those 37% fibers were within MFD of 30–35 µm, indicating an optimal distribution similar to uninjured group. Taken together, muscle weight reduction, histology and histomorphometric analysis revealed that stepwise cut nerve transection resulted in lesser muscle atrophy.

### Functional recovery following transection injury

**Figure 7:**
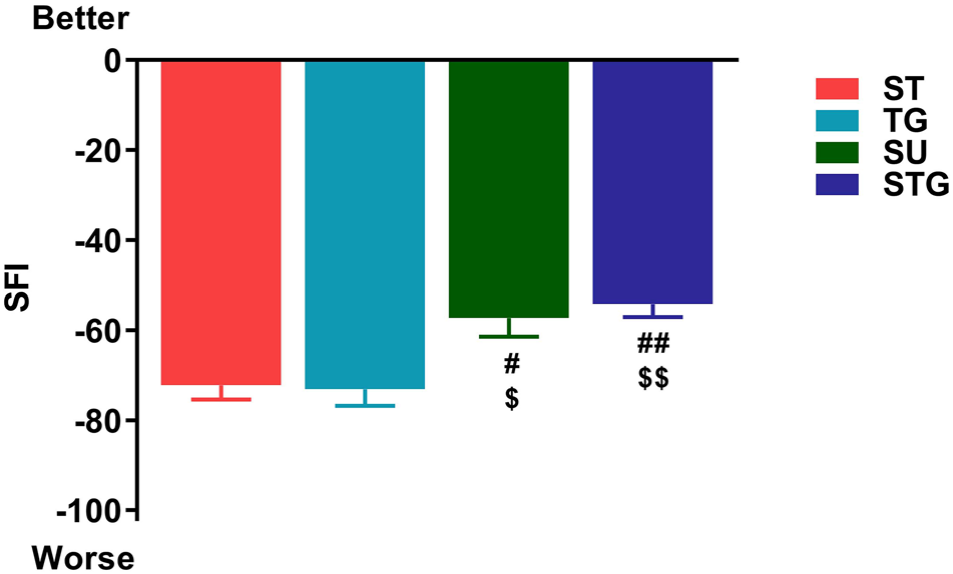
Effect of different transection models on the functional recovery (SFI) at post-injury day 28. n = 10–12/group. ^#^*P* < 0.05, ^##^*P* < 0.01 vs. ST, and ^$^*P* < 0.05, ^$$^*P* < 0.01 vs. TG. For other details, see Fig. 8.

We found that four transection methods differentially affected the architectural structures of nerves, neural microvessels, and innervated muscles. To evaluate how the changes in transected nerve, blood vessels and muscle atrophy modulate the functional recovery, SFI was determined at post-injury day 28. Figure 7 shows that SFI recovery was directly related to the extent of nerve misdirection, angiogenesis, and muscle atrophy. Mice with STG had significantly better functional recovery (− 54.12 ± 2.91) compared with ST (− 72.16 ± 3.16) and TG (− 73.05 ± 3.69) groups and it was comparable to SU (− 57.21 ± 4.16) group. The better functional recovery following nerve transection with minimal gap suggests that close approximation of the severed nerve ends and subsequent coordinated favorable neuromusculovascular processes are crucial in alleviating nerve transection-induced functional deficit.

## Discussion

The main finding of this study is that our novel stepwise nerve transection with glue (STG) is an easily reproducible method with minimal gap formation and without additional nerve manipulation compared to nerve transection and epineural sutures. Our results with four different methods of nerve transection methods clearly suggest that it is the gap distance between the severed nerve ends that determines the extent of regenerating axons’ directional growth, neuroangiogenesis, denervation induced muscle atrophy, and functional recovery. The novel stepwise nerve transection method with glue thus presents a reliable model to investigate nerve transection-induced complex morphological, cellular, molecular, and functional changes and interactions that occur in between nerves, vessels, and muscles.

A complex multicellular event takes place after nerve transection. First, the new tissue known as the bridge composed of Schwann cell, inflammatory cells, perineurial cells, fibroblasts and extracellular matrix is formed in a poorly understood cell–cell interaction between proximal and distal stumps^26–28^. Axons distal to transection site undergo Wallerian degeneration. Second, once the bridge is formed, cords of Schwann cells pass into and across the bridge, and guide the regenerating axons across this expanse of new tissue^27,28^. Regeneration of transected nerve is a slow process and the severed nerve with wider gap takes longer time to be connected with the end organ^8^. The therapeutic failure with nerve repair often results from misdirection of regenerating axons to functionally inappropriate end organs^11,12,16^. In our ST and TG methods, the nerve was completely transected and the gap caused by elastic retraction of severed nerve ends was spontaneous and uncontrolled. To prevent further displacement of the severed nerve ends in TG method, fibrin glue was applied to the transection site. In contrast, transection-induced nerve gaps were controlled in SU and STG groups by sutures or glue, respectively. Among all methods, ST had the highest whereas SU and STG groups had the lowest misaligned nerve fibers in the proximal and distal zones of regenerating axons. However, in the injury zone, STG group had the lowest misaligned fibers compared to all groups. Our findings with ST are consistent with a recent study where whole mount imaging of mouse sciatic nerve transection injury demonstrated an extensive axon guidance defect around the nerve bridge with large populations of axons growing along the outside of both the proximal and distal nerve ends^24^. In agreement with the negative impact of misdirected nerve fibers in nerve regeneration and functional recovery^11,12,16^, qualitative and quantitative analysis of transected nerves in our study demonstrate that closer approximation and minimum manipulation of the severed nerve ends are associated with reduced misdirection of axonal regrowth.

Angiogenesis following an injury plays an important role in nerve regeneration because of nutritional and tropic factors provided by the vascular system^17,29,30^. Schwann cells use the newly formed blood vessels as tracks to guide the regeneration of axon with the right direction across the nerve bridge^26,27^. Recently, whole mount imaging of mouse sciatic nerve demonstrated CD31 positive new blood vessel formation in the proximal and distal stumps of the transected nerve^24^. Consistent with this imaging study, we further confirmed neural angiogenesis by quantitative analysis. Our quantitative analysis demonstrated a significantly increased blood vessel formation in the proximal and injury zones of transected nerve with minimal gap (SU and STG groups) than in the transected nerves with wider gaps (ST and TG groups). Importantly, new blood vessels in SU and STG groups were optimally maintained in the distal ends. It is thus possible that the minimum gap length in SU and STG provide a favorable microenvironment for directional angiogenesis, which can guide Schwann cells and re-growing axons into the distal stump.

The structure and function of skeletal muscle are directly regulated by motor nerves, and it is well known that denervation of the muscles leads to significant contractile deficits and rapid muscle-fiber atrophy^14,15,25^. The denervated muscle is characterized by reduced muscle mass, decreased CSA, increased connective tissue, increased fibrosis, and reduced metabolic capacity^31,32^, which can be reversed by timely reinnervation^15,33^. Muscle wet weight is widely used to evaluate muscle innervation^15,34–36^. Our interesting findings with nerves and neural blood vessels were well reflected in the gross muscle weight and histomorphometric analysis of the denervated muscles. We observed significantly less muscle atrophy in transection groups with a standardized minimized gap than in the transection groups with wider gaps. Moreover, myofiber diameter and distribution were significantly better in STG group compared to all other groups. The overall findings of this study not only demonstrate the extent of relationship between nerves, blood vessels, and muscles following different types of transection, but reaffirm that a close approximation of the severed nerve ends are associated with reduced muscle atrophy and thus an improved functional recovery.

While it is seldom possible to duplicate a human disease condition in animals because animal models often recover differently than humans, our work was sparked by a dearth of literature where the injury is reproducibly controlled and the functional outcomes are rigorously measured. Experimental models of peripheral nerve injury are widely used to investigate the intricate mechanisms of nerve regeneration and the benefits novel therapeutic strategies. Most commonly used models in rodents are nerve crush and nerve transection injuries. While crush injury recovers well over weeks, the absence of a reproducible and clinically relevant experimental model of nerve transection with epineural suture continues to impede our efforts to better understand, diagnose, and treat these injuries. Almost all of the rodent peripheral nerve transection models employ sharp transection of the entire nerve using scissors and most, but not all, involve an immediate repair. In the clinic, the severed ends of a transected nerve are freshly cut for better fascicular bundle visualization, apposition and repair with tensionless epineurial sutures. We devised the stepwise technique to see what the maximum effect of minimizing the gap and misalignment could have on outcome. Perhaps because of the variable manipulation of nerve ends required for tension free nerve repairs, the outcomes of direct nerve repair are highly dependent on circumstances, such as operator skill and variable effects of trauma. Therefore, by taking the advantage of clinically employed fibrin glue, we were able to develop and validate a novel nerve transection model in mice that has a close resemblance to the characteristics of the suture model with regard to transected nerves, neural microvessels, innervated muscles, and post-injury functional recovery. Our novel method, the stepwise cut and fibrin glue technique has several advantages as an experimental model when compared with gold standard end-to-end neurorrhaphy, including: (i) standardized gap distance without suturing, (ii) simplicity and easiness with the procedure, (iii) reproducibility with faster procedure time, and (iv) lack of confounding factors associated with surgeon manipulation.

For the successful evaluation of the efficacy of a novel treatment strategy, it is essential to have a simple and reproducible pre-clinical animal model. We have successfully established a novel nerve transection model in mice by using fibrin glue. This transection method decreases the surgical difficulties and variability by avoiding microsurgical manipulations on the nerve, and thus ensures the reproducibility and reliability of the animal model. Although it is quite impossible to exactly mimic the pathophysiology seen with transection with sutures, we hope that the close resemblance of our STG model with SU that we described can be easily reproduced by any lab and the data generated by this method would significantly contribute to better understand the nerve pathophysiology and molecular mechanisms of nerve regeneration, and the development of novel strategies for nerve regeneration and functional recovery.

In conclusions, limited data exist on how each method of nerve repair might influence the morphological and functional aspects of nerve recovery in relation to the injuries. Specifically no previous work rigorously separates the issues of inadvertent gap formation, suture repair manipulation (gripping the nerve multiple times), and orientation of the nerve stumps with precision. It is in this light that we most wish for our result to be interpreted. The novelty of this study is therefore not in the fact that we see misdirection of axons, or vessel outgrowth per se as reported by others^24,26,30^. Rather, the novelty is that quantification of misdirection and vessel growth in our model is free of three critical confounders: Inadvertent gaps, gripping the nerve multiple times for suturing, and mis-orientation of the nerve stumps. None of the previous studies allows this interpretation because of no explicit demonstration of gap-free and fixed orientation repairs in the literature^22,24,26,30,37^, and our STG model provides a clearer picture of these processes in the most optimal, gap-free, and unmanipulated condition. We think that is important for the study of nerve repair, and will be clinically relevant in future work where treatments are compared to controls in these well-controlled settings.

## Materials and methods

### Animals

The experimental design and animal protocols were approved by the Institutional Animal Care and Use Committee (IACUC) at Penn State University College of Medicine. Forty two 10-week-old male C57BL/6J mice (Jackson Laboratories, Bar Harbor, ME) weighing 20–25 g were used in this study (Supplementary Table 1). The mice were housed at the animal facility and the experimental animals were handled according to the IACUC guidelines for the care and use of laboratory animals.

### Mouse models of peripheral nerve transection injury

**Figure 8:**
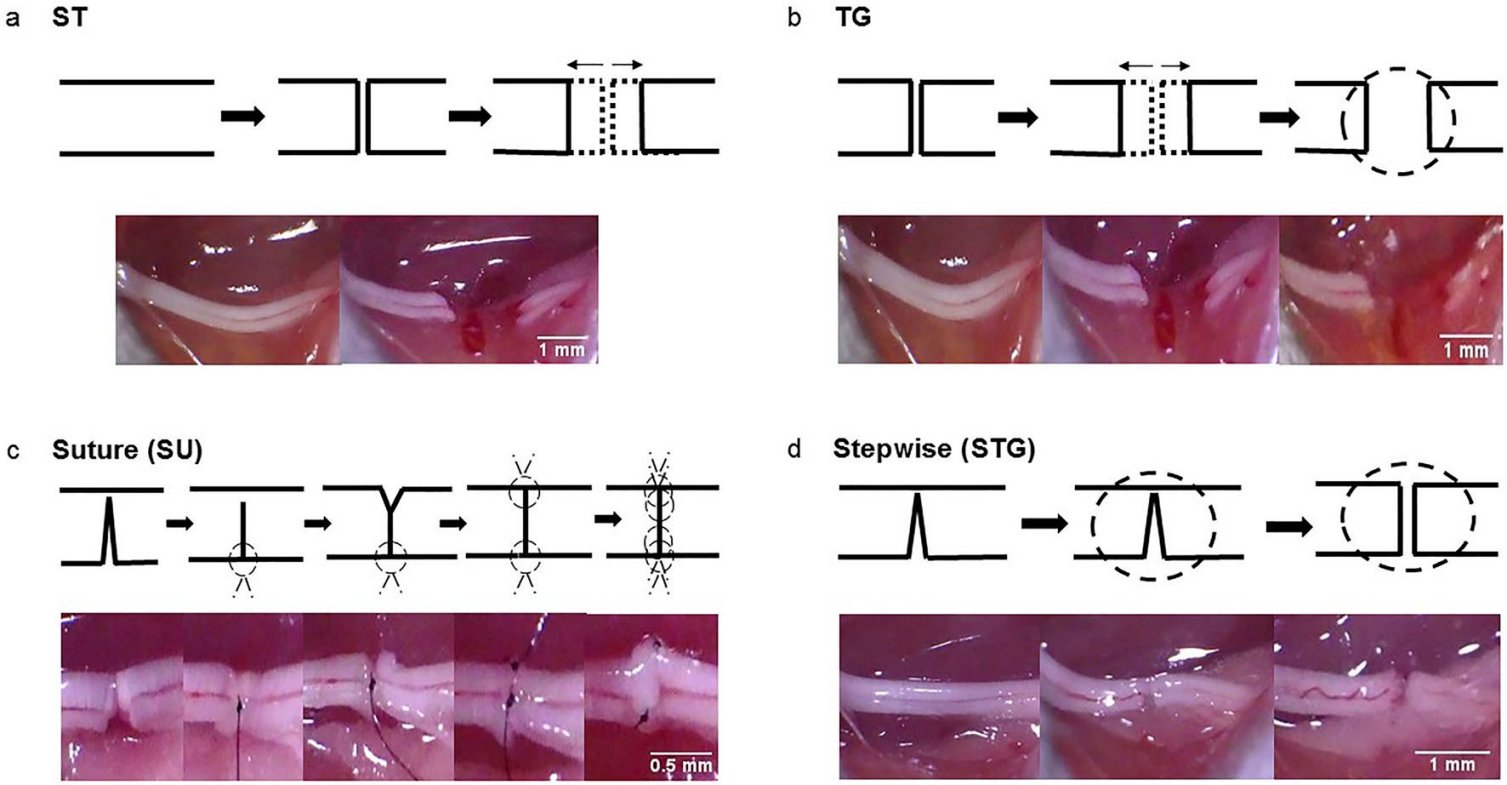
Experimental groups with schematic surgical steps (top panel) and representative gross nerve images (lower panel) of surgical procedures in different nerve transection methods. Experimental groups are (**a**) Simple Transection (ST), (**b**) Transection & Glue (TG), (**c**) Stepwise Transection and Sutures (SU), and (**d**) Stepwise Transection and Glue (STG). The complete transection of the nerve was confirmed under direct microscopic vision.

The mice were anesthetized with intraperitoneal ketamine (60 mg/kg) and xylazine (4 mg/kg) anesthesia, right hind limb and lower back were shaved, washed with 70% ethanol, and prepped with povidone iodine. A 2 cm long skin incision was made on the extended posterior right hind limb to carefully expose the right sciatic nerve through trans-gluteal approach under an operating microscope. Extreme care was taken to avoid any iatrogenic mechanical damage to the sciatic nerve. Mice were randomly assigned into four groups for four different types of nerve transection (Fig. 8). The end points of this study were analyzed after 4 weeks to note early changes associated with these repair methods.

In *Simple Transection *(*ST*) method (Fig. 8a), the nerve was completely transected in one attempt using fine microscissor (Integra Miltex, Plainsboro, NJ) at 2 mm proximal to the trifurcation of sciatic nerve. This transection method is widely used as a model of severe nerve injury to investigate the regeneration process where the nerve gap caused by spontaneous elastic retraction of the cut nerve ends is uncontrolled and arbitrary. In *Transection & Glue *(*TG*) method (Fig. 8b), the nerve was completely transected similar to the *ST* group and the nerve gap was also uncontrolled and arbitrary, but 20 µl of fibrin glue (TISSEEL, Baxter, Deerfield, IL) was applied around the transection site to limit any further displacement of the severed nerve ends. In *Stepwise Transection and Sutures *(*SU*) method (Fig. 8c), the nerve was incompletely cut to 80% of its width to prevent gap formation, then the lacerated ends of nerve were repaired by epineural suture using one stich of 9-0 nylon. Then, the remaining 20% of the nerve was completely transected and repaired using one stich of 9-0 nylon. After flipping over the nerve, the posterior surface of nerve was repaired using two stiches of 9-0 nylon. In *Stepwise Transection and Glue *(*STG*) method (Fig. 8d), the nerve was incompletely cut to 80% of its width to prevent the gap formation. Then, 20 µl of fibrin glue was applied around the laceration site and the remaining 20% of the nerve was completely transected before the complete clotting of the glue. This method not only effectively minimized gap formation caused by elastic retraction of the cut nerve ends, it was also free from additional injury and manipulation caused by suturing. In all models, complete transection was confirmed under direct microscopic evaluation by a trained microsurgeon. The skin was closed by surgical staples and post-operative slow release buprenorphine (0.05 mg/kg) was given subcutaneously to all animals as an analgesic. Animals were returned to their cages and monitored on the warming pad until active. Animals were then returned to the animal facility, allowed free activity, and followed under the supervision of the attending veterinarian. The surgical staples were removed on post-surgery day 14 and functional analysis was performed at specific time points.

### Sciatic function index (SFI) as determined by walking track analysis

Walking track analysis was performed as previously described to evaluate the direct in vivo global motor function recovery^38,39^. Briefly, mice were trained to walk freely along a 77 cm by 7 cm corridor lined with white paper and individual footprint of the hind limbs were obtained by painting each foot with ink before surgery and on post-surgery day 28. At least three measurable footprints for each hind limb were obtained. Two blinded observers selected three footprints per hind limb, which were measured by digital calipers. SFI was calculated using three parameters of footprints by two blinded observers to the study groups: (1) toe spread (TS, first through fifth toes), (2) total print length (PL), and (3) intermediate toe spread (IT, second, third and fourth toes), and the following formula: SFI = − 38.3 [(EPL − NPL)/NPL] + 109.5 [(ETS − NTS)/NTS] + 13.3 [(EIT − NIT)/NIT] − 8.8^39^, where E is the experimental paw and N is the normal paw. In general, an index of 0 − 10 indicates normal function and an index of − 100 represents complete loss of function.

### Whole mount immunostaining of nerves

The whole mount nerve immunostaining was performed as described by Dun XP^24^. Briefly, after SFI analysis on post-surgery day 28, nerves were collected and fixed for 5 h in 4% paraformaldehyde at 4 °C. Grossly, the nerves were found in well-connected condition and in good continuity with a bulge at the repair site, and there was no dehiscence after 28 days post-surgery. Nerves were washed three times 10 min each with PTX {(phosphate buffered saline (PBS) with 1% Triton X-100 (Sigma X100))} and incubated in blocking solution (10% normal goat serum (Jackson Immunoresearch, 005-000-121) in 5% BSA PTX) overnight at 4 °C. Next day, Nerves were transferred into primary antibodies in 5% BSA PTX and incubated for 72 h at 4 °C with gentle rocking. Primary antibodies were neurofilament heavy chain (NF-H) (1:1000, Novus biologicals, NB300-135), P-zero Myelin protein (MP0) (1:500, Aves Labs, PZ0), and CD31 (1:100, BD Pharmingen, 553370). Nerves were then washed with PTX for 4 h at 4 °C, with a change of PTX every 1 h. After PTX washes, nerves were incubated with Alexa Fluor 488, 594 and 647-conjugated secondary antibodies (1:500, Invitrogen) for 48 h at 4 °C with gentle rocking. Nerves were washed in PTX three times for 15 min each, followed by 4-h washing in PTX with PTX change at every hour. Nerves were then washed overnight without changing PTX at 4 °C. Next day, nerves were washed with PBS (three times 10 min each) for the removal of triton and cleared sequentially in 25%, 50% glycerol (Sigma, G6279) in PBS for 6 and 12 h respectively for each glycerol concentration. Following clearing, nerves were mounted in SlowFade Gold Antifade Mountant with DAPI (Invitrogen, S36939) or without DAPI (Invitrogen, S36937). Stained whole nerves were imaged using ZEISS Axio Observer 7 equipped with an Apotome.2 (Carl Zeiss Microscopy GmbH, Jena, Germany). Tiling and Z-stack functions both were employed to image whole nerve. Maximum Intensity projection was used to pull the data from all Z-stacks and represented as 2D image.

### Quantitative analysis of nerve fibers and blood vessels

Maximum Intensity projected 2-D images were used for the quantification of horizontal and vertical fibers. Briefly, Nerve images were captured at different depths using Z-stack imaging. Data from different depths (3D-data) was pulled together as 2D-Image using maximum intensity projection. Use of maximum intensity projected 2D-image nullifies possibility of recounting same fiber at different depths. The bulging in the nerve was the transection site with a nerve bridge^24,40^ and we termed this as the injury zone. For the purpose of quantification, the imaged whole nerve was divided into 3 zones: Proximal, injury and distal. All nerve regions immediately before and after the bulge were proximal and distal zones, respectively. Each zone was then divided into grid using Image J. The number of horizontal and vertical fibers were calculated in every grid manually.

The division of each zone into many grids was performed to ease manual counting. The percentage of horizontal and vertical fibers were then calculated from the data. Three nerves from different animals were analyzed per group. Directional Image analysis (degree of orientation) of nerve fibers was done using Orientation J plugin from Image J (National Institutes of Health, Bethesda, MD)^41^. Analysis of vessel density and vessel branching were done using AngioTool version 0.6a (02.18.14), an open source windows executable software which provides automated measures of vessel area and number of junctions^42^.

### Hematoxylin and eosin (H&E) staining and histomorphometric analysis of muscles

After functional analysis on post-surgery day 28, tibialis anterior (TA) muscles of contralateral and injured hind limbs were harvested from surrounding tissues and weighed. H&E staining and histomorphometric analysis of TA muscles were performed as described previously^43^. Briefly, muscles were washed in chilled PBS and immediately transferred to 30% sucrose and kept at 4 °C until tissue sank to bottom. Muscles were embedded in OCT tissue freezing medium (Sakura Torrance, USA) in dry ice isopentane (2-methyl butane) slurry. 10 μm thick sections were cut at − 21 °C using a Microm HM505e Cryostat and collected on superfrost plus microscopic slides (Fisherbrand). Slides were then stained for H&E and imaged on Olympus BX53 microscope. Quantitative analysis for cross sectional area (CSA), minimum Feret’s diameter (MFD), and fiber distribution of muscle fiber was done with Image J software. Three random microscopic fields were chosen from each muscle and 3 muscles from different animals were analyzed per group.

### Data analysis

All results are presented as means ± SEM and all experiments were repeated at least three times. Data were analyzed using GraphPad PRISM 7 (GraphPad Software, San Diego, CA, USA) and statistical analysis were performed using one-way ANOVA followed by Tukey’s post-hoc test for multiple comparisons. A *P* value of < 0.05 was considered to be statistically significant.

## Supplementary Information

Supplementary Information.
