# Supplementary material for: A novel nerve transection and repair method in mice: histomorphometric analysis of nerves, blood vessels, and muscles with functional recovery
Source: Sci Rep. 2020 Dec 10;10:21637. doi: 10.1038/s41598-020-78481-1 (PMC7729850; doi:10.1038/s41598-020-78481-1)

A novel nerve transection and repair method in mice: Histomorphometric analysis of nerves, blood vessels, and muscles with functional recovery

Jung Il Lee^1,2*^, Anagha A. Gurjar^1*^, M A Hassan Talukder^1#^, Andrew Rodenhouse^1^, Kristen Manto^1^, Mary O’Brien^1^, Prem Kumar Govindappa,^1^ and John C. Elfar^1#^

^1^Department of Orthopaedics and Rehabilitation, Center for Orthopaedics Research and Translational Science, The Pennsylvania State University College of Medicine, Milton S. Hershey Medical Center, Hershey, Pennsylvania, USA; ^2^Department of Orthopedic Surgery, Hanyang University College of Medicine, Hayang University Guri Hospital, South Korea

**Supplementary Figure 1**


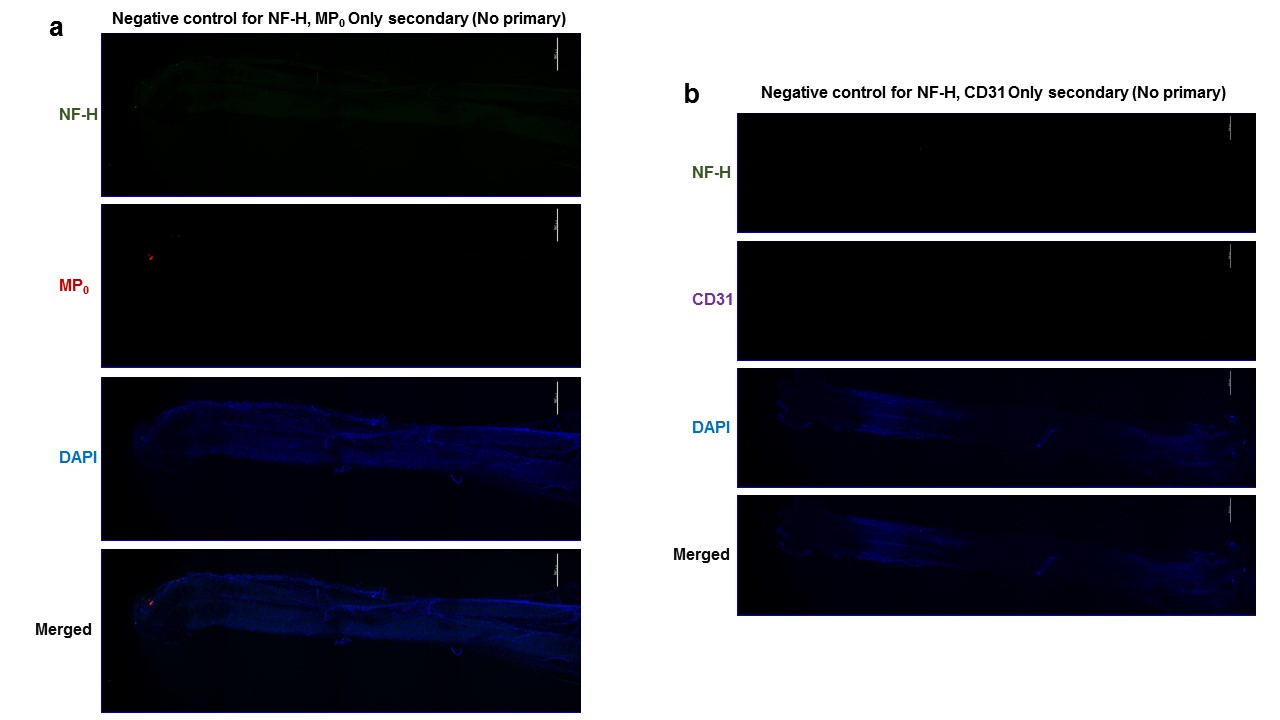


**Legend for Supplementary Figure 1.** Representative compact images for the negative control of immunofluorescence staining of the whole nerves. (**a**) NF-H, MP0, DAPI and merged. (**b**) NF-H, CD31, DAPI and merged. Scale bar, 500 µm at upper right corner; magnification, 5x.

**Supplementary Table 1.** Number of animals used in each model and in each experiment. Experimental groups are Simple Transection (ST), Transection & Glue (TG), Stepwise Transection and Sutures (SU), and Stepwise Transection and Glue (STG).


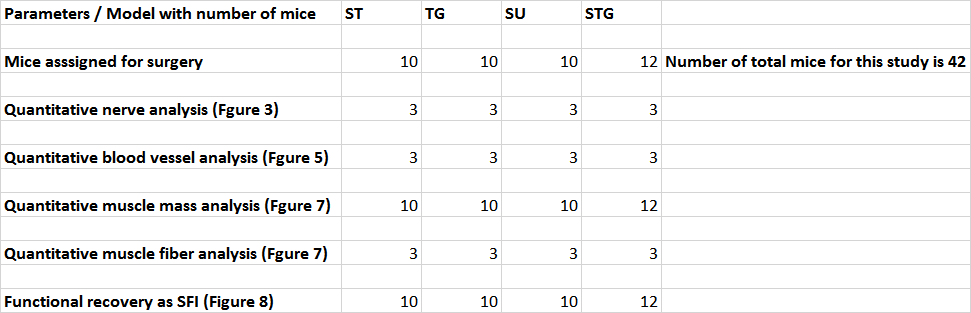

Supplement: Supplementary file 1 — Supplementary Information. [file 41598_2020_78481_MOESM1_ESM.docx]
